# Supplementary figures and images for: A predictive model of asymmetric morphogenesis from 3D reconstructions of mouse heart looping dynamics
Source: eLife. 2017 Nov 28;6:e28951. doi: 10.7554/eLife.28951 (PMC5705212; doi:10.7554/eLife.28951)

VENTRAL VIEW

DORSAL VIEW

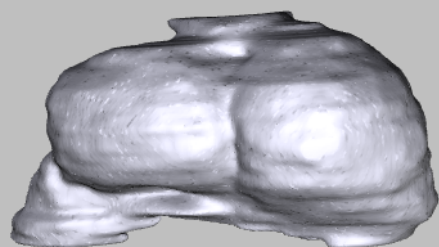

E8.5e

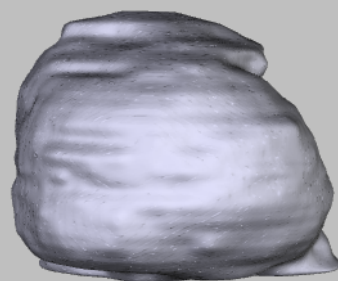

E8.5f

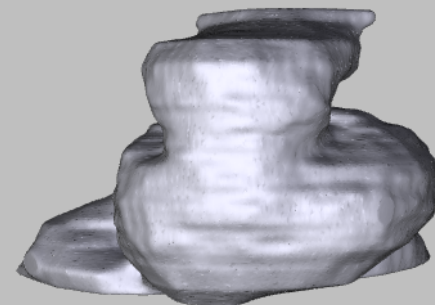

E8.5g

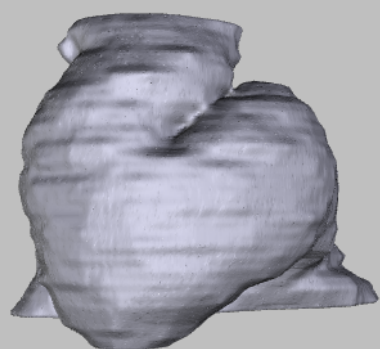

E8.5h

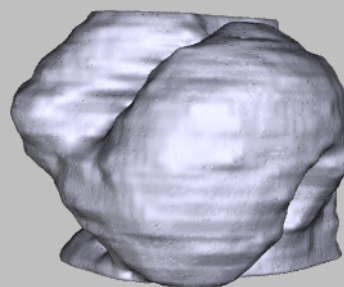

E8.5i

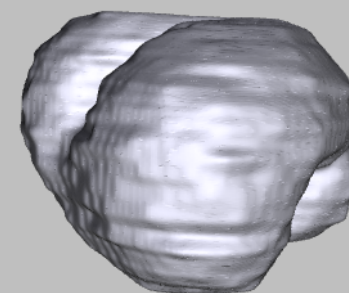

E8.5j

Supplement: Figure 1—source data 1. — 3D visualisation of the heart, as reconstructed from HREM images, at the six stages shown in Figure 1. This file should be opened with Adobe Acrobat Reader. Click on the image to activate the manual rotation of the reconstruction. Each image may be rotated at will with the mouse (hold left click). Zoom in and out with the mouse wheel. Shortcuts at the top align all images in a ventral or dorsal view, with the notochord vertical. [file elife-28951-fig1-data1.pdf]
