# Supplementary material for: A predictive model of asymmetric morphogenesis from 3D reconstructions of mouse heart looping dynamics
Source: eLife. 2017 Nov 28;6:e28951. doi: 10.7554/eLife.28951 (PMC5705212; doi:10.7554/eLife.28951)
Supplement: Figure 9—source data 1. — Ventral (left) and dorsal (right) views are shown, aligned with the notochord vertical (green). The myocardial layer (yellow) is made transparent, revealing the tube axis (red). Samples are identified with a number (S). The length of the heart tube is indicated, as well as the somite number (So) of the corresponding embryo. The genotype of control samples is Shh+/+ (S11, S17, S20) or Shh+/- (S8, S14). Scale bar: 100 µm. [file elife-28951-fig9-data1.pptx]

## Slide 1
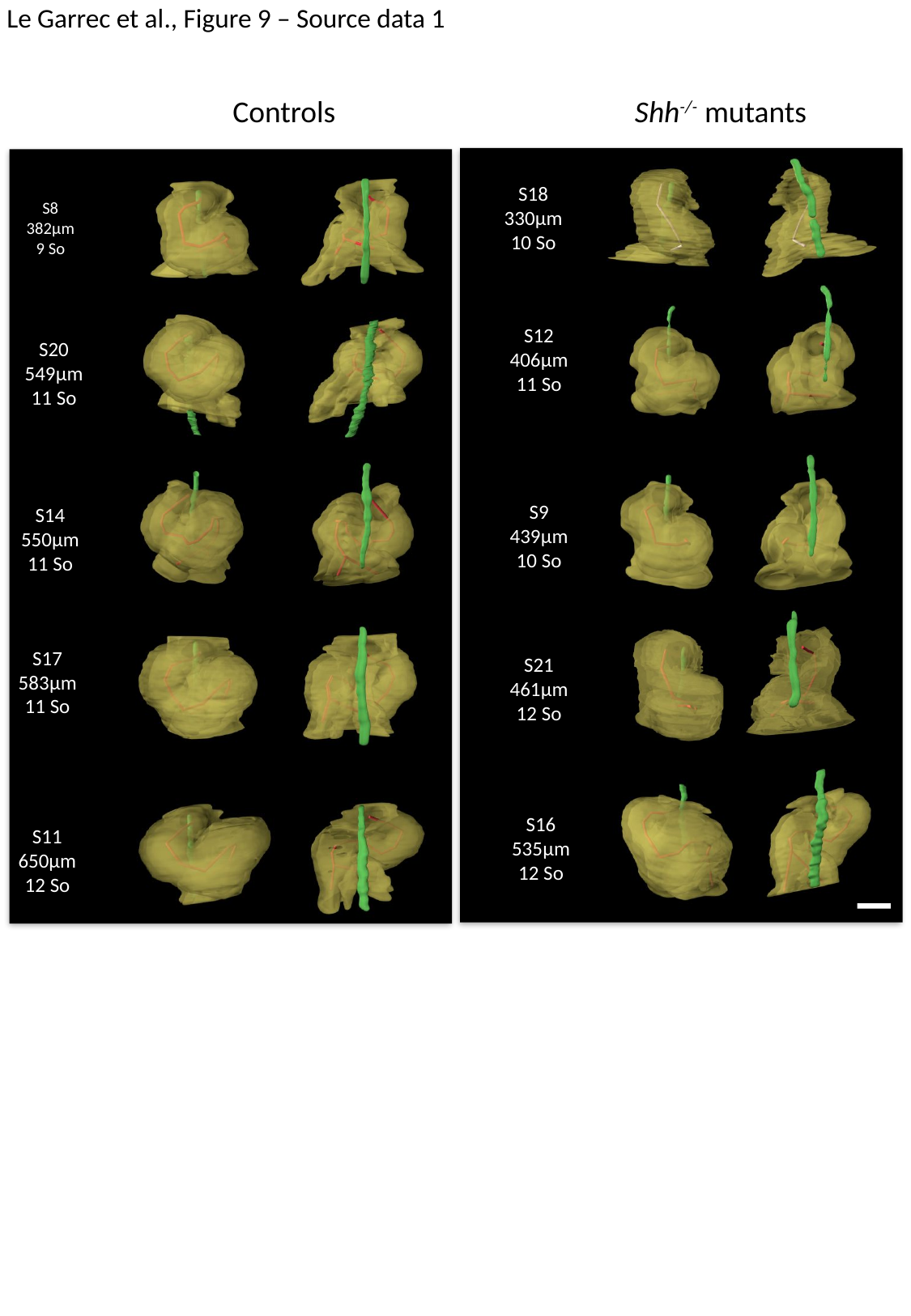

Le Garrec et al., Figure 9 – Source data 1
Controls
Shh-/- mutants
S8
382µm
9 So
S18
330µm
10 So
S12
406μm
11 So
S20
549µm
11 So
S14
550µm
11 So
S9
439μm
10 So
S17
583μm
11 So
S21
461μm
12 So
S16
535μm
12 So
S11
650µm
12 So
