# Supplementary material for: A predictive model of asymmetric morphogenesis from 3D reconstructions of mouse heart looping dynamics
Source: eLife. 2017 Nov 28;6:e28951. doi: 10.7554/eLife.28951 (PMC5705212; doi:10.7554/eLife.28951)
Supplement: Source code 2. [file elife-28951-code2.docx]

**Source code 2 : base model**

function m = gpt_source_code_2_base_model_20170512( m )

%m = gpt_source_code_1_base_model_20170512( m )

% Morphogen interaction function.

% Written at 2017-05-12 09:23:02.

% GFtbox revision 5411, 2016-02-26 12:00.

% The user may edit any part of this function between delimiters

% of the form "USER CODE..." and "END OF USER CODE...". The

% delimiters themselves must not be moved, edited, deleted, or added.

if isempty(m), return; end

fprintf( 1, '%s found in %s\n', mfilename(), which(mfilename()) );

try

m = local_setproperties( m );

catch

end

setGlobals();

realtime = m.globalDynamicProps.currenttime;

dt = m.globalProps.timestep;

%%% USER CODE: INITIALISATION

% In this section you may modify the mesh in any way whatsoever.

%%% END OF USER CODE: INITIALISATION

%%% SECTION 1: ACCESSING MORPHOGENS AND TIME.

%%% AUTOMATICALLY GENERATED CODE: DO NOT EDIT.

polariser_i = FindMorphogenRole( m, 'POLARISER' );

P = m.morphogens(:,polariser_i);

[kapar_i,kapar_p,kapar_a,kapar_l] = getMgenLevels( m, 'KAPAR' );

[kaper_i,kaper_p,kaper_a,kaper_l] = getMgenLevels( m, 'KAPER' );

[kbpar_i,kbpar_p,kbpar_a,kbpar_l] = getMgenLevels( m, 'KBPAR' );

[kbper_i,kbper_p,kbper_a,kbper_l] = getMgenLevels( m, 'KBPER' );

[knor_i,knor_p,knor_a,knor_l] = getMgenLevels( m, 'KNOR' );

[strainret_i,strainret_p,strainret_a,strainret_l] = getMgenLevels( m, 'STRAINRET' );

[arrest_i,arrest_p,arrest_a,arrest_l] = getMgenLevels( m, 'ARREST' );

[id_venous_pole_i,id_venous_pole_p,id_venous_pole_a,id_venous_pole_l] = getMgenLevels( m, 'ID_VENOUS_POLE' );

[id_arterial_pole_i,id_arterial_pole_p,id_arterial_pole_a,id_arterial_pole_l] = getMgenLevels( m, 'ID_ARTERIAL_POLE' );

[id_right_ventricle_i,id_right_ventricle_p,id_right_ventricle_a,id_right_ventricle_l] = getMgenLevels( m, 'ID_RIGHT_VENTRICLE' );

[id_left_ventricle_i,id_left_ventricle_p,id_left_ventricle_a,id_left_ventricle_l] = getMgenLevels( m, 'ID_LEFT_VENTRICLE' );

[id_oft_i,id_oft_p,id_oft_a,id_oft_l] = getMgenLevels( m, 'ID_OFT' );

[id_atria_i,id_atria_p,id_atria_a,id_atria_l] = getMgenLevels( m, 'ID_ATRIA' );

[id_art_band_i,id_art_band_p,id_art_band_a,id_art_band_l] = getMgenLevels( m, 'ID_ART_BAND' );

[id_vein_band_i,id_vein_band_p,id_vein_band_a,id_vein_band_l] = getMgenLevels( m, 'ID_VEIN_BAND' );

[id_iv_band_i,id_iv_band_p,id_iv_band_a,id_iv_band_l] = getMgenLevels( m, 'ID_IV_BAND' );

[id_dorsal_line_i,id_dorsal_line_p,id_dorsal_line_a,id_dorsal_line_l] = getMgenLevels( m, 'ID_DORSAL_LINE' );

[id_ventral_line_i,id_ventral_line_p,id_ventral_line_a,id_ventral_line_l] = getMgenLevels( m, 'ID_VENTRAL_LINE' );

[id_right_i,id_right_p,id_right_a,id_right_l] = getMgenLevels( m, 'ID_RIGHT' );

[id_left_i,id_left_p,id_left_a,id_left_l] = getMgenLevels( m, 'ID_LEFT' );

[id_inner_curvature_i,id_inner_curvature_p,id_inner_curvature_a,id_inner_curvature_l] = getMgenLevels( m, 'ID_INNER_CURVATURE' );

[id_outer_curvature_i,id_outer_curvature_p,id_outer_curvature_a,id_outer_curvature_l] = getMgenLevels( m, 'ID_OUTER_CURVATURE' );

[id_sdorsal_line_i,id_sdorsal_line_p,id_sdorsal_line_a,id_sdorsal_line_l] = getMgenLevels( m, 'ID_SDORSAL_LINE' );

[id_sleft_i,id_sleft_p,id_sleft_a,id_sleft_l] = getMgenLevels( m, 'ID_SLEFT' );

[id_siv_i,id_siv_p,id_siv_a,id_siv_l] = getMgenLevels( m, 'ID_SIV' );

[s_iv_i,s_iv_p,s_iv_a,s_iv_l] = getMgenLevels( m, 'S_IV' );

[s_dorsal_line_i,s_dorsal_line_p,s_dorsal_line_a,s_dorsal_line_l] = getMgenLevels( m, 'S_DORSAL_LINE' );

[s_left_i,s_left_p,s_left_a,s_left_l] = getMgenLevels( m, 'S_LEFT' );

[s_art_band_i,s_art_band_p,s_art_band_a,s_art_band_l] = getMgenLevels( m, 'S_ART_BAND' );

[id_sart_band_i,id_sart_band_p,id_sart_band_a,id_sart_band_l] = getMgenLevels( m, 'ID_SART_BAND' );

[s_vein_band_i,s_vein_band_p,s_vein_band_a,s_vein_band_l] = getMgenLevels( m, 'S_VEIN_BAND' );

[id_svein_band_i,id_svein_band_p,id_svein_band_a,id_svein_band_l] = getMgenLevels( m, 'ID_SVEIN_BAND' );

[s_ventral_line_i,s_ventral_line_p,s_ventral_line_a,s_ventral_line_l] = getMgenLevels( m, 'S_VENTRAL_LINE' );

[id_sventral_line_i,id_sventral_line_p,id_sventral_line_a,id_sventral_line_l] = getMgenLevels( m, 'ID_SVENTRAL_LINE' );

[s_right_i,s_right_p,s_right_a,s_right_l] = getMgenLevels( m, 'S_RIGHT' );

[id_sright_i,id_sright_p,id_sright_a,id_sright_l] = getMgenLevels( m, 'ID_SRIGHT' );

[id_ventral_left_i,id_ventral_left_p,id_ventral_left_a,id_ventral_left_l] = getMgenLevels( m, 'ID_VENTRAL_LEFT' );

[s_outer_curvature_i,s_outer_curvature_p,s_outer_curvature_a,s_outer_curvature_l] = getMgenLevels( m, 'S_OUTER_CURVATURE' );

[id_souter_curvature_i,id_souter_curvature_p,id_souter_curvature_a,id_souter_curvature_l] = getMgenLevels( m, 'ID_SOUTER_CURVATURE' );

[s_ventral_left_i,s_ventral_left_p,s_ventral_left_a,s_ventral_left_l] = getMgenLevels( m, 'S_VENTRAL_LEFT' );

[id_sventral_left_i,id_sventral_left_p,id_sventral_left_a,id_sventral_left_l] = getMgenLevels( m, 'ID_SVENTRAL_LEFT' );

[s_inner_curvature_i,s_inner_curvature_p,s_inner_curvature_a,s_inner_curvature_l] = getMgenLevels( m, 'S_INNER_CURVATURE' );

[id_sinner_curvature_i,id_sinner_curvature_p,id_sinner_curvature_a,id_sinner_curvature_l] = getMgenLevels( m, 'ID_SINNER_CURVATURE' );

[id_dorsal_right_i,id_dorsal_right_p,id_dorsal_right_a,id_dorsal_right_l] = getMgenLevels( m, 'ID_DORSAL_RIGHT' );

[s_dorsal_right_i,s_dorsal_right_p,s_dorsal_right_a,s_dorsal_right_l] = getMgenLevels( m, 'S_DORSAL_RIGHT' );

[id_sdorsal_right_i,id_sdorsal_right_p,id_sdorsal_right_a,id_sdorsal_right_l] = getMgenLevels( m, 'ID_SDORSAL_RIGHT' );

[s_oft_i,s_oft_p,s_oft_a,s_oft_l] = getMgenLevels( m, 'S_OFT' );

[id_soft_i,id_soft_p,id_soft_a,id_soft_l] = getMgenLevels( m, 'ID_SOFT' );

[s_atria_i,s_atria_p,s_atria_a,s_atria_l] = getMgenLevels( m, 'S_ATRIA' );

[id_satria_i,id_satria_p,id_satria_a,id_satria_l] = getMgenLevels( m, 'ID_SATRIA' );

[id_clone_density_i,id_clone_density_p,id_clone_density_a,id_clone_density_l] = getMgenLevels( m, 'ID_CLONE_DENSITY' );

[s_arterial_pole_i,s_arterial_pole_p,s_arterial_pole_a,s_arterial_pole_l] = getMgenLevels( m, 'S_ARTERIAL_POLE' );

[s_venous_pole_i,s_venous_pole_p,s_venous_pole_a,s_venous_pole_l] = getMgenLevels( m, 'S_VENOUS_POLE' );

[id_left_side_i,id_left_side_p,id_left_side_a,id_left_side_l] = getMgenLevels( m, 'ID_LEFT_SIDE' );

[id_right_side_i,id_right_side_p,id_right_side_a,id_right_side_l] = getMgenLevels( m, 'ID_RIGHT_SIDE' );

[id_sarterial_pole_i,id_sarterial_pole_p,id_sarterial_pole_a,id_sarterial_pole_l] = getMgenLevels( m, 'ID_SARTERIAL_POLE' );

[s_right_ventricle_i,s_right_ventricle_p,s_right_ventricle_a,s_right_ventricle_l] = getMgenLevels( m, 'S_RIGHT_VENTRICLE' );

[id_sright_ventricle_i,id_sright_ventricle_p,id_sright_ventricle_a,id_sright_ventricle_l] = getMgenLevels( m, 'ID_SRIGHT_VENTRICLE' );

[id_oft_band_i,id_oft_band_p,id_oft_band_a,id_oft_band_l] = getMgenLevels( m, 'ID_OFT_BAND' );

[id_soft_band_i,id_soft_band_p,id_soft_band_a,id_soft_band_l] = getMgenLevels( m, 'ID_SOFT_BAND' );

[s_oft_band_i,s_oft_band_p,s_oft_band_a,s_oft_band_l] = getMgenLevels( m, 'S_OFT_BAND' );

% Mesh type: cylinder

% basecap: 0

% baseheight: 1

% baserings: 0

% circumdivs: 30

% height: 1.5

% heightdivs: 30

% radius: 0.1

% randomness: 0

% topcap: 0

% topheight: 1

% toprings: 0

% version: 1

% Morphogen Diffusion Decay Dilution Mutant

% --------------------------------------------------

% KAPAR ---- ---- ---- ----

% KAPER ---- ---- ---- ----

% KBPAR ---- ---- ---- ----

% KBPER ---- ---- ---- ----

% KNOR ---- ---- ---- ----

% POLARISER 0.05 ---- ---- ----

% STRAINRET ---- ---- ---- ----

% ARREST ---- ---- ---- ----

% ID_VENOUS_POLE ---- ---- ---- ----

% ID_ARTERIAL_POLE ---- ---- ---- ----

% ID_RIGHT_VENTRICLE ---- ---- ---- ----

% ID_LEFT_VENTRICLE ---- ---- ---- ----

% ID_OFT ---- ---- ---- ----

% ID_ATRIA ---- ---- ---- ----

% ID_ART_BAND ---- ---- ---- ----

% ID_VEIN_BAND ---- ---- ---- ----

% ID_IV_BAND ---- ---- ---- ----

% ID_DORSAL_LINE ---- ---- ---- ----

% ID_VENTRAL_LINE ---- ---- ---- ----

% ID_RIGHT ---- ---- ---- ----

% ID_LEFT ---- ---- ---- ----

% ID_INNER_CURVATURE ---- ---- ---- ----

% ID_OUTER_CURVATURE ---- ---- ---- ----

% ID_SDORSAL_LINE ---- ---- ---- ----

% ID_SLEFT ---- ---- ---- ----

% ID_SIV ---- ---- ---- ----

% S_IV 0.0001 0.1 ---- ----

% S_DORSAL_LINE 0.0001 0.1 ---- ----

% S_LEFT 0.0001 0.1 ---- ----

% S_ART_BAND 0.0001 0.1 ---- ----

% ID_SART_BAND ---- ---- ---- ----

% S_VEIN_BAND 0.0001 0.1 ---- ----

% ID_SVEIN_BAND ---- ---- ---- ----

% S_VENTRAL_LINE 0.0001 0.1 ---- ----

% ID_SVENTRAL_LINE ---- ---- ---- ----

% S_RIGHT 0.0001 0.1 ---- ----

% ID_SRIGHT ---- ---- ---- ----

% ID_VENTRAL_LEFT ---- ---- ---- ----

% S_OUTER_CURVATURE 0.0001 0.1 ---- ----

% ID_SOUTER_CURVATURE ---- ---- ---- ----

% S_VENTRAL_LEFT 0.0001 0.1 ---- ----

% ID_SVENTRAL_LEFT ---- ---- ---- ----

% S_INNER_CURVATURE 0.0001 0.1 ---- ----

% ID_SINNER_CURVATURE ---- ---- ---- ----

% ID_DORSAL_RIGHT ---- ---- ---- ----

% S_DORSAL_RIGHT 0.0001 0.1 ---- ----

% ID_SDORSAL_RIGHT ---- ---- ---- ----

% S_OFT 0.0001 0.1 ---- ----

% ID_SOFT ---- ---- ---- ----

% S_ATRIA 0.0001 0.1 ---- ----

% ID_SATRIA ---- ---- ---- ----

% ID_CLONE_DENSITY ---- ---- ---- ----

% S_ARTERIAL_POLE 0.01 0.05 ---- ----

% S_VENOUS_POLE 0.0001 0.1 ---- ----

% ID_LEFT_SIDE ---- ---- ---- ----

% ID_RIGHT_SIDE ---- ---- ---- ----

% ID_SARTERIAL_POLE ---- ---- ---- ----

% S_RIGHT_VENTRICLE 0.0001 0.1 ---- ----

% ID_SRIGHT_VENTRICLE ---- ---- ---- ----

% ID_OFT_BAND ---- ---- ---- ----

% ID_SOFT_BAND ---- ---- ---- ----

% S_OFT_BAND ---- 0.1 ---- ----

%%% USER CODE: MORPHOGEN INTERACTIONS

% In this section you may modify the mesh in any way that does not

% alter the set of nodes.

% initial constraints at the poles and along the dorsal mesocardium (DM)

m=leaf_fix_vertex(m,'vertex',[],'dfs','');

m=leaf_fix_vertex(m,'vertex',38:30:878,'dfs','xyz');% all vertices of the DM are fixed in xyz

m=leaf_fix_vertex(m,'vertex',39:30:879,'dfs','xyz');% all vertices of the DM are fixed in xyz

m=leaf_fix_vertex(m,'vertex',908:909,'dfs','xyz');% attachment of arterial pole (allowing rotation)

m=leaf_fix_vertex(m,'vertex',1:30,'dfs','xyz');% all vertices of the venous pole are fixed in xyz

m=leaf_fix_vertex(m,'vertex',901:907,'dfs','z');% all vertices of the arterial pole are fixed in z

m=leaf_fix_vertex(m,'vertex',910:930,'dfs','z');% all vertices of the arterial pole are fixed in z

if Steps(m)==0

% Put any code here that should only be performed at the start of

% the simulation, for example, to set up initial morphogen values.

% geometry

maxz=max(m.nodes(:,3)); % the 3 stands for z axis, i.e. x,y,z are 1,2,3

minz=min(m.nodes(:,3));

epsilon=0.02;

id_venous_pole_p(m.nodes(:,3)<minz+epsilon)=1;

id_arterial_pole_p(m.nodes(:,3)>maxz-epsilon)=1;

d=maxz-minz;

top=d*0.77;

bot=d*0.62;

id_right_ventricle_p((m.nodes(:,3)>bot+minz)&(m.nodes(:,3)<top+minz))=1;

top=d*0.70;

bot=d*0.20;

id_left_ventricle_p((m.nodes(:,3)>bot+minz)&(m.nodes(:,3)<top+minz))=1;

top=d*0.85;

bot=d*0.75;

id_oft_p((m.nodes(:,3)>bot+minz)&(m.nodes(:,3)<top+minz))=1;

top=d*0.50;

bot=d*0.00;

id_atria_p((m.nodes(:,3)>minz)&(m.nodes(:,3)<top+minz))=1;

top=d*0.62;

bot=d*0.60;

id_iv_band_p((m.nodes(:,3)>bot+minz)&(m.nodes(:,3)<top+minz))=1;

top=d*0.87;

bot=d*0.62;

id_art_band_p((m.nodes(:,3)>bot+minz)&(m.nodes(:,3)<top+minz))=1;

top=d*0.60;

bot=d*0.35;

id_vein_band_p((m.nodes(:,3)>bot+minz)&(m.nodes(:,3)<top+minz))=1;

top=d*1.0;

bot=d*0.70;

id_oft_band_p((m.nodes(:,3)>bot+minz)&(m.nodes(:,3)<top+minz))=1;

dorsal=max(m.nodes(:,2));

id_dorsal_line_p(m.nodes(:,2)>dorsal-epsilon/4)=1;

ventral=min(m.nodes(:,2));

id_ventral_line_p(m.nodes(:,2)<ventral+epsilon/4)=0.5; % was 1

left=max(m.nodes(:,1));

right=min(m.nodes(:,1));

id_right_p(m.nodes(:,1)<right+epsilon/4)=1;

id_left_p(m.nodes(:,1)>left-epsilon/4)=1;

% inner curvature = dorsal_left

id_inner_curvature_p((m.nodes(:,1)>(left*sqrt(2)/2)-epsilon) &(m.nodes(:,2)>(dorsal*sqrt(2)/2)-epsilon))=1;

% outer curvature = ventral_right

id_outer_curvature_p((m.nodes(:,1)<(right*sqrt(2)/2)+epsilon) &(m.nodes(:,2)<(ventral*sqrt(2)/2)+epsilon))=1;

% ventral_left

id_ventral_left_p((m.nodes(:,1)>(left*sqrt(2)/2)-epsilon) &(m.nodes(:,2)<(ventral*sqrt(2)/2)+epsilon))=1;

% dorsal_right

id_dorsal_right_p((m.nodes(:,1)<(right*sqrt(2)/2)+1*epsilon) &(m.nodes(:,2)>(dorsal*sqrt(2)/2)-1*epsilon))=1;% was 1*epsilon

% left and right sides

id_left_side_p(m.nodes(:,1)>0)=1;

id_right_side_p(m.nodes(:,1)<0)=1;

end

% m = leaf_setstrainpolarisation(m, 'mode', 'compression', 'scaling', -5, 'proportional', true, 'useanisotropy', false);

m = leaf_setproperty( m, 'useGrowthTensors', true, 'useMorphogens', true );

m = leaf_setstrainpolarisation(m, 'mode', 'compression', 'scaling', 5, 'proportional', true, 'useanisotropy', true);

if (realtime>10-dt) && (realtime<10+dt)

% morphogen gradients established at t=10

id_sdorsal_line_p=1.*s_dorsal_line_l;

id_sventral_line_p=1.*s_ventral_line_l;

id_sleft_p =1.*s_left_l;

id_sright_p =1.*s_right_l;

id_sart_band_p =1.*s_art_band_l;

id_svein_band_p =1.*s_vein_band_l;

id_soft_p =1.*s_oft_l;

id_satria_p =1.*s_atria_l;

id_siv_p =1.0.*s_iv_l;

id_souter_curvature_p =1.0.*s_outer_curvature_l;

id_sinner_curvature_p =1.0.*s_inner_curvature_l;

id_sventral_left_p =1.0.*s_ventral_left_l;

id_sdorsal_right_p =1.0.*s_dorsal_right_l;

id_sarterial_pole_p =1.0.*s_arterial_pole_l;

id_sright_ventricle_p =1.0.*s_right_ventricle_l;

id_soft_band_p =1.0.*s_oft_band_l;

elseif (realtime>10+dt)&& (realtime<20)% was 30

BASICGROWTH=0.02;

kaper_p=6.0*BASICGROWTH* (id_left_side_p - id_right_side_p).*s_arterial_pole_l...% rotation at the arterial pole

+ 0.7*BASICGROWTH*id_svein_band_p...% Left Ventricle (LV) inflation

+ 0.40*BASICGROWTH*id_sright_ventricle_p.*inh(100,id_sinner_curvature_p);% Right Ventricle (RV) inflation

kbper_p=6.0*BASICGROWTH* (id_left_side_p - id_right_side_p).*s_arterial_pole_l...% rotation at the arterial pole

+ 0.7*BASICGROWTH*id_svein_band_p...% LV inflation

+ 0.40*BASICGROWTH*id_sright_ventricle_p.*inh(100,id_sinner_curvature_p);% RV inflation

kapar_p=BASICGROWTH * (5.0.*id_siv_p.*inh(100,id_sdorsal_line_p));% ventral bending

kbpar_p=BASICGROWTH * (1.0);

elseif (realtime>=20)&& (realtime<30)

m=leaf_fix_vertex(m,'vertex',[],'dfs','');

m=leaf_fix_vertex(m,'vertex',38:30:338,'dfs','xyz');% progressive breakdown of DM

m=leaf_fix_vertex(m,'vertex',39:30:339,'dfs','xyz');% progressive breakdown of DM

m=leaf_fix_vertex(m,'vertex',578:30:878,'dfs','xyz');% progressive breakdown of DM

m=leaf_fix_vertex(m,'vertex',579:30:879,'dfs','xyz');% progressive breakdown of DM

m=leaf_fix_vertex(m,'vertex',908:909,'dfs','xyz');% attachment of arterial pole (allowing rotation)

m=leaf_fix_vertex(m,'vertex',1:30,'dfs','xyz');% all vertices of the venous pole are fixed in xyz

m=leaf_fix_vertex(m,'vertex',901:907,'dfs','z');% all vertices of the arterial pole are fixed in z

m=leaf_fix_vertex(m,'vertex',910:930,'dfs','z');% all vertices of the arterial pole are fixed in z

BASICGROWTH=0.02;

kaper_p=6.0*BASICGROWTH* (id_left_side_p - id_right_side_p).*s_arterial_pole_l...% rotation at the arterial pole

+ 0.7*BASICGROWTH*id_svein_band_p...% LV inflation

+ 0.40*BASICGROWTH*id_sright_ventricle_p.*inh(100,id_sinner_curvature_p);% RV inflation

kbper_p=6.0*BASICGROWTH* (id_left_side_p - id_right_side_p).*s_arterial_pole_l...% rotation at the arterial pole

+ 0.7*BASICGROWTH*id_svein_band_p...% LV inflation

+ 0.40*BASICGROWTH*id_sright_ventricle_p.*inh(100,id_sinner_curvature_p);% RV inflation

kapar_p=BASICGROWTH * (5.0.*id_siv_p.*inh(100,id_sdorsal_line_p));% ventral bending

kbpar_p=BASICGROWTH * (1.0);

elseif (realtime>=30)&& (realtime<40)

m=leaf_fix_vertex(m,'vertex',[],'dfs','');

m=leaf_fix_vertex(m,'vertex',38:30:278,'dfs','yz');% progressive breakdown of DM

m=leaf_fix_vertex(m,'vertex',39:30:279,'dfs','yz');% progressive breakdown of DM

m=leaf_fix_vertex(m,'vertex',638:30:878,'dfs','yz');% progressive breakdown of DM

m=leaf_fix_vertex(m,'vertex',639:30:879,'dfs','yz');% progressive breakdown of DM

m=leaf_fix_vertex(m,'vertex',908:909,'dfs','xyz');% attachment of arterial pole (allowing rotation)

m=leaf_fix_vertex(m,'vertex',1:30,'dfs','xyz');% all vertices of the venous pole are fixed in xyz

m=leaf_fix_vertex(m,'vertex',901:907,'dfs','z');% all vertices of the arterial pole are fixed in z

m=leaf_fix_vertex(m,'vertex',910:930,'dfs','z');% all vertices of the arterial pole are fixed in z

BASICGROWTH=0.02;

kaper_p=0.004 ...

+ 0.7*BASICGROWTH*id_svein_band_p...% LV inflation

+ 0.40*BASICGROWTH*id_sright_ventricle_p.*inh(100,id_sinner_curvature_p);% RV inflation

kbper_p=0.004 ...

+ 0.7*BASICGROWTH*id_svein_band_p...% LV inflation

+ 0.40*BASICGROWTH*id_sright_ventricle_p.*inh(100,id_sinner_curvature_p);% RV inflation

kapar_p=BASICGROWTH * (5.0.*id_siv_p.*inh(100,id_sdorsal_line_p)...% ventral bending

+ 4.0*(4*s_venous_pole_l).*id_satria_p.*inh(100,id_sleft_p));% venous pole asymmetry

kbpar_p=BASICGROWTH * (1.0...

+ 4.0*(4*s_venous_pole_l).*id_satria_p.*inh(100,id_sleft_p));% venous pole asymmetry

elseif (realtime>=40)&& (realtime<50)

m=leaf_fix_vertex(m,'vertex',[],'dfs','');

m=leaf_fix_vertex(m,'vertex',38:30:218,'dfs','yz');% progressive breakdown of DM

m=leaf_fix_vertex(m,'vertex',39:30:219,'dfs','yz');% progressive breakdown of DM

m=leaf_fix_vertex(m,'vertex',698:30:878,'dfs','yz');% progressive breakdown of DM

m=leaf_fix_vertex(m,'vertex',699:30:879,'dfs','yz');% progressive breakdown of DM

m=leaf_fix_vertex(m,'vertex',908:909,'dfs','xyz');% attachment of arterial pole (allowing rotation)

m=leaf_fix_vertex(m,'vertex',1:30,'dfs','xyz');% all vertices of the venous pole are fixed in xyz

m=leaf_fix_vertex(m,'vertex',901:907,'dfs','z');% all vertices of the arterial pole are fixed in z

m=leaf_fix_vertex(m,'vertex',910:930,'dfs','z');% all vertices of the arterial pole are fixed in z

BASICGROWTH=0.02;

kaper_p=0.004 ...

+ 0.7*BASICGROWTH*id_svein_band_p...% LV inflation

+ 0.40*BASICGROWTH*id_sright_ventricle_p.*inh(100,id_sinner_curvature_p);% RV inflation

kbper_p=0.004 ...

+ 0.7*BASICGROWTH*id_svein_band_p...% LV inflation

+ 0.40*BASICGROWTH*id_sright_ventricle_p.*inh(100,id_sinner_curvature_p);% RV inflation

kapar_p=BASICGROWTH * (1.3...

+ 4.0*(4*s_venous_pole_l).*id_satria_p.*inh(100,id_sleft_p));% venous pole asymmetry

kbpar_p=BASICGROWTH * (1.3...

+ 4.0*(4*s_venous_pole_l).*id_satria_p.*inh(100,id_sleft_p));% venous pole asymmetry

elseif (realtime>=50)&& (realtime<60)

m=leaf_fix_vertex(m,'vertex',[],'dfs','');

m=leaf_fix_vertex(m,'vertex',38:30:158,'dfs','yz');% progressive breakdown of DM

m=leaf_fix_vertex(m,'vertex',39:30:159,'dfs','yz');% progressive breakdown of DM

m=leaf_fix_vertex(m,'vertex',758:30:878,'dfs','yz');% progressive breakdown of DM

m=leaf_fix_vertex(m,'vertex',759:30:879,'dfs','yz');% progressive breakdown of DM

m=leaf_fix_vertex(m,'vertex',908:909,'dfs','xyz');% attachment of arterial pole

m=leaf_fix_vertex(m,'vertex',1:30,'dfs','xyz');% all vertices of the venous pole are fixed in xyz

m=leaf_fix_vertex(m,'vertex',901:907,'dfs','yz');% all vertices of the arterial pole are fixed in yz

m=leaf_fix_vertex(m,'vertex',910:930,'dfs','yz');% all vertices of the arterial pole are fixed in yz

BASICGROWTH=0.02;

kaper_p=0.004 ...

+ 0.7*BASICGROWTH*id_svein_band_p...% LV inflation

+ 0.40*BASICGROWTH*id_sright_ventricle_p.*inh(100,id_sinner_curvature_p);% RV inflation

kbper_p=0.004 ...

+ 0.7*BASICGROWTH*id_svein_band_p...% LV inflation

+ 0.40*BASICGROWTH*id_sright_ventricle_p.*inh(100,id_sinner_curvature_p);% RV inflation

kapar_p=BASICGROWTH * (1.3...

+ 4.0*(4*s_venous_pole_l).*id_satria_p.*inh(100,id_sleft_p));% venous pole asymmetry

kbpar_p=BASICGROWTH * (1.3...

+ 4.0*(4*s_venous_pole_l).*id_satria_p.*inh(100,id_sleft_p));% venous pole asymmetry

elseif (realtime>=60)&& (realtime<70)

m=leaf_fix_vertex(m,'vertex',[],'dfs','');

m=leaf_fix_vertex(m,'vertex',38:30:98,'dfs','yz');% progressive breakdown of DM

m=leaf_fix_vertex(m,'vertex',39:30:99,'dfs','yz');% progressive breakdown of DM

m=leaf_fix_vertex(m,'vertex',818:30:878,'dfs','yz');% progressive breakdown of DM

m=leaf_fix_vertex(m,'vertex',819:30:879,'dfs','yz');% progressive breakdown of DM

m=leaf_fix_vertex(m,'vertex',908:909,'dfs','xyz');% attachment of arterial pole

m=leaf_fix_vertex(m,'vertex',1:30,'dfs','xyz');% all vertices of the venous pole are fixed in xyz

m=leaf_fix_vertex(m,'vertex',901:907,'dfs','yz');% all vertices of the arterial pole are fixed in yz

m=leaf_fix_vertex(m,'vertex',910:930,'dfs','yz');% all vertices of the arterial pole are fixed in yz

BASICGROWTH=0.02;

kaper_p=0.008 ...

+ 0.7*BASICGROWTH*id_svein_band_p...% LV inflation

+ 0.4*BASICGROWTH*id_sart_band_p.*inh(100,id_sinner_curvature_p);% RV inflation

kbper_p=0.008 ...

+ 0.7*BASICGROWTH*id_svein_band_p...% LV inflation

+ 0.4*BASICGROWTH*id_sart_band_p.*inh(100,id_sinner_curvature_p);% RV inflation

kapar_p=BASICGROWTH * (1.3);

kbpar_p=BASICGROWTH * (1.3);

elseif (realtime>=70)&& (realtime<80)

m=leaf_fix_vertex(m,'vertex',[],'dfs','');

m=leaf_fix_vertex(m,'vertex',38:30:38,'dfs','yz');% progressive breakdown of DM

m=leaf_fix_vertex(m,'vertex',39:30:39,'dfs','yz');% progressive breakdown of DM

m=leaf_fix_vertex(m,'vertex',878:30:878,'dfs','yz');% progressive breakdown of DM

m=leaf_fix_vertex(m,'vertex',879:30:879,'dfs','yz');% progressive breakdown of DM

m=leaf_fix_vertex(m,'vertex',908:909,'dfs','xyz');% attachment of arterial pole

m=leaf_fix_vertex(m,'vertex',1:30,'dfs','xyz');% all vertices of the venous pole are fixed in xyz

m=leaf_fix_vertex(m,'vertex',901:907,'dfs','yz');% all vertices of the arterial pole are fixed in yz

m=leaf_fix_vertex(m,'vertex',910:930,'dfs','yz');% all vertices of the arterial pole are fixed in yz

BASICGROWTH=0.02;

kaper_p=0.008 ...

+ 0.7*BASICGROWTH*id_svein_band_p...% LV inflation

+ 0.4*BASICGROWTH*id_sart_band_p.*inh(100,id_sinner_curvature_p);% RV inflation

kbper_p=0.008 ...

+ 0.7*BASICGROWTH*id_svein_band_p...% LV inflation

+ 0.4*BASICGROWTH*id_sart_band_p.*inh(100,id_sinner_curvature_p);% RV inflation

kapar_p=BASICGROWTH * (1.3);

kbpar_p=BASICGROWTH * (1.3);

elseif (realtime>=80)&& (realtime<120)

m=leaf_fix_vertex(m,'vertex',[],'dfs','');

m=leaf_fix_vertex(m,'vertex',908:909,'dfs','xyz');% attachment of arterial pole

m=leaf_fix_vertex(m,'vertex',1:30,'dfs','xyz');% all vertices of the venous pole are fixed in xyz

m=leaf_fix_vertex(m,'vertex',901:907,'dfs','yz');% all vertices of the arterial pole are fixed in yz

m=leaf_fix_vertex(m,'vertex',910:930,'dfs','yz');% all vertices of the arterial pole are fixed in yz

BASICGROWTH=0.02;

kaper_p=0.008 ...

+ 0.7*BASICGROWTH*id_svein_band_p...% LV inflation

+ 0.4*BASICGROWTH*id_sart_band_p.*inh(100,id_sinner_curvature_p);% RV inflation

kbper_p=0.008 ...

+ 0.7*BASICGROWTH*id_svein_band_p...% LV inflation

+ 0.4*BASICGROWTH*id_sart_band_p.*inh(100,id_sinner_curvature_p);% RV inflation

kapar_p=BASICGROWTH * (1.3);

kbpar_p=BASICGROWTH * (1.3);

end

m.mgen_production(:,polariser_i) = 0.1...

*(1.0*id_venous_pole_p...

+ 1.0 ...

- P.*(id_arterial_pole_l));% set polarizer gradient between source and sink

m.mgen_production(:,s_iv_i) = 0.1*(id_iv_band_p);

m.mgen_production(:,s_art_band_i) = 0.1*(id_art_band_p);

m.mgen_production(:,s_vein_band_i) = 0.1*id_vein_band_p;

m.mgen_production(:,s_oft_i) = 0.1*id_oft_p;

m.mgen_production(:,s_atria_i) = 0.1*id_atria_p;

m.mgen_production(:,s_dorsal_line_i) = 0.1*(id_dorsal_line_p);

m.mgen_production(:,s_ventral_line_i) = 0.1*(id_ventral_line_p);

m.mgen_production(:,s_left_i) = 0.1*(id_left_p);

m.mgen_production(:,s_right_i) = 0.1*(id_right_p);

m.mgen_production(:,s_outer_curvature_i) = 0.1*(id_outer_curvature_p);

m.mgen_production(:,s_inner_curvature_i) = 0.1*(id_inner_curvature_p);

m.mgen_production(:,s_ventral_left_i) = 0.1*(id_ventral_left_p);

m.mgen_production(:,s_dorsal_right_i) = 0.1*(id_dorsal_right_p);

m.mgen_production(:,s_arterial_pole_i) = 0.1*(id_arterial_pole_p);

m.mgen_production(:,s_venous_pole_i) = 0.1*(id_venous_pole_p);

m.mgen_production(:,s_right_ventricle_i) = 0.1*(id_right_ventricle_p);

m.mgen_production(:,s_oft_band_i) = 0.1*(id_oft_band_p);

%%% END OF USER CODE: MORPHOGEN INTERACTIONS

%%% SECTION 3: INSTALLING MODIFIED VALUES BACK INTO MESH STRUCTURE

%%% AUTOMATICALLY GENERATED CODE: DO NOT EDIT.

m.morphogens(:,polariser_i) = P;

m.morphogens(:,kapar_i) = kapar_p;

m.morphogens(:,kaper_i) = kaper_p;

m.morphogens(:,kbpar_i) = kbpar_p;

m.morphogens(:,kbper_i) = kbper_p;

m.morphogens(:,knor_i) = knor_p;

m.morphogens(:,strainret_i) = strainret_p;

m.morphogens(:,arrest_i) = arrest_p;

m.morphogens(:,id_venous_pole_i) = id_venous_pole_p;

m.morphogens(:,id_arterial_pole_i) = id_arterial_pole_p;

m.morphogens(:,id_right_ventricle_i) = id_right_ventricle_p;

m.morphogens(:,id_left_ventricle_i) = id_left_ventricle_p;

m.morphogens(:,id_oft_i) = id_oft_p;

m.morphogens(:,id_atria_i) = id_atria_p;

m.morphogens(:,id_art_band_i) = id_art_band_p;

m.morphogens(:,id_vein_band_i) = id_vein_band_p;

m.morphogens(:,id_iv_band_i) = id_iv_band_p;

m.morphogens(:,id_dorsal_line_i) = id_dorsal_line_p;

m.morphogens(:,id_ventral_line_i) = id_ventral_line_p;

m.morphogens(:,id_right_i) = id_right_p;

m.morphogens(:,id_left_i) = id_left_p;

m.morphogens(:,id_inner_curvature_i) = id_inner_curvature_p;

m.morphogens(:,id_outer_curvature_i) = id_outer_curvature_p;

m.morphogens(:,id_sdorsal_line_i) = id_sdorsal_line_p;

m.morphogens(:,id_sleft_i) = id_sleft_p;

m.morphogens(:,id_siv_i) = id_siv_p;

m.morphogens(:,s_iv_i) = s_iv_p;

m.morphogens(:,s_dorsal_line_i) = s_dorsal_line_p;

m.morphogens(:,s_left_i) = s_left_p;

m.morphogens(:,s_art_band_i) = s_art_band_p;

m.morphogens(:,id_sart_band_i) = id_sart_band_p;

m.morphogens(:,s_vein_band_i) = s_vein_band_p;

m.morphogens(:,id_svein_band_i) = id_svein_band_p;

m.morphogens(:,s_ventral_line_i) = s_ventral_line_p;

m.morphogens(:,id_sventral_line_i) = id_sventral_line_p;

m.morphogens(:,s_right_i) = s_right_p;

m.morphogens(:,id_sright_i) = id_sright_p;

m.morphogens(:,id_ventral_left_i) = id_ventral_left_p;

m.morphogens(:,s_outer_curvature_i) = s_outer_curvature_p;

m.morphogens(:,id_souter_curvature_i) = id_souter_curvature_p;

m.morphogens(:,s_ventral_left_i) = s_ventral_left_p;

m.morphogens(:,id_sventral_left_i) = id_sventral_left_p;

m.morphogens(:,s_inner_curvature_i) = s_inner_curvature_p;

m.morphogens(:,id_sinner_curvature_i) = id_sinner_curvature_p;

m.morphogens(:,id_dorsal_right_i) = id_dorsal_right_p;

m.morphogens(:,s_dorsal_right_i) = s_dorsal_right_p;

m.morphogens(:,id_sdorsal_right_i) = id_sdorsal_right_p;

m.morphogens(:,s_oft_i) = s_oft_p;

m.morphogens(:,id_soft_i) = id_soft_p;

m.morphogens(:,s_atria_i) = s_atria_p;

m.morphogens(:,id_satria_i) = id_satria_p;

m.morphogens(:,id_clone_density_i) = id_clone_density_p;

m.morphogens(:,s_arterial_pole_i) = s_arterial_pole_p;

m.morphogens(:,s_venous_pole_i) = s_venous_pole_p;

m.morphogens(:,id_left_side_i) = id_left_side_p;

m.morphogens(:,id_right_side_i) = id_right_side_p;

m.morphogens(:,id_sarterial_pole_i) = id_sarterial_pole_p;

m.morphogens(:,s_right_ventricle_i) = s_right_ventricle_p;

m.morphogens(:,id_sright_ventricle_i) = id_sright_ventricle_p;

m.morphogens(:,id_oft_band_i) = id_oft_band_p;

m.morphogens(:,id_soft_band_i) = id_soft_band_p;

m.morphogens(:,s_oft_band_i) = s_oft_band_p;

%%% USER CODE: FINALISATION

% In this section you may modify the mesh in any way whatsoever.

%%% END OF USER CODE: FINALISATION

end

%%% USER CODE: SUBFUNCTIONS

function m = local_setproperties( m )

% This function is called at time zero in the INITIALISATION section of the

% interaction function. It provides commands to set each of the properties

% that are contained in m.globalProps. Uncomment whichever ones you would

% like to set yourself, and put in whatever value you want.

%

% Some of these properties are for internal use only and should never be

% set by the user. At some point these will be moved into a different

% component of m, but for the present, just don't change anything unless

% you know what it is you're changing.

% m = leaf_setproperty( m, 'trinodesvalid', true );

% m = leaf_setproperty( m, 'prismnodesvalid', true );

% m = leaf_setproperty( m, 'thicknessRelative', 0.100000 );

% m = leaf_setproperty( m, 'thicknessArea', 0.000000 );

% m = leaf_setproperty( m, 'activeGrowth', 1.000000 );

% m = leaf_setproperty( m, 'displayedGrowth', 1 );

% m = leaf_setproperty( m, 'allowNegativeGrowth', true );

% m = leaf_setproperty( m, 'usePrevDispAsEstimate', true );

% m = leaf_setproperty( m, 'mingradient', 0.000000 );

% m = leaf_setproperty( m, 'thresholdsq', 0.005874 );

% m = leaf_setproperty( m, 'splitmargin', 1.000000 );

% m = leaf_setproperty( m, 'thresholdmgen', 0.500000 );

% m = leaf_setproperty( m, 'bulkmodulus', 1.000000 );

% m = leaf_setproperty( m, 'poissonsRatio', 0.300000 );

% m = leaf_setproperty( m, 'timestep', 2.500000 );

% m = leaf_setproperty( m, 'timeunitname', '' );

% m = leaf_setproperty( m, 'distunitname', 'mm' );

% m = leaf_setproperty( m, 'validateMesh', true );

% m = leaf_setproperty( m, 'allowSplitLongFEM', false );

% m = leaf_setproperty( m, 'longSplitThresholdPower', 0.000000 );

% m = leaf_setproperty( m, 'allowSplitBentFEM', false );

% m = leaf_setproperty( m, 'allowSplitBio', true );

% m = leaf_setproperty( m, 'allowFlipEdges', false );

% m = leaf_setproperty( m, 'allowElideEdges', false );

% m = leaf_setproperty( m, 'mincellangle', 0.200000 );

% m = leaf_setproperty( m, 'alwaysFlat', 0.000000 );

% m = leaf_setproperty( m, 'flatten', false );

% m = leaf_setproperty( m, 'flattenratio', 1.000000 );

m = leaf_setproperty( m, 'useGrowthTensors', true, 'useMorphogens', true );

% m = leaf_setproperty( m, 'plasticGrowth', false );

% m = leaf_setproperty( m, 'totalinternalrotation', 1.239019 );

% m = leaf_setproperty( m, 'stepinternalrotation', 2.000000 );

% m = leaf_setproperty( m, 'showinternalrotation', false );

% m = leaf_setproperty( m, 'internallyrotated', false );

% m = leaf_setproperty( m, 'maxFEcells', 0.000000 );

% m = leaf_setproperty( m, 'inittotalcells', 0.000000 );

% m = leaf_setproperty( m, 'maxBioAcells', 0.000000 );

% m = leaf_setproperty( m, 'maxBioBcells', 0.000000 );

% m = leaf_setproperty( m, 'colors', (3 values) );

% m = leaf_setproperty( m, 'colorvariation', 1.000000 );

% m = leaf_setproperty( m, 'colorparams', (6 values) );

% m = leaf_setproperty( m, 'freezing', 0.000000 );

% m = leaf_setproperty( m, 'mgen_interaction', (unknown type ''function_handle'') );

% m = leaf_setproperty( m, 'mgen_interactionName', 'heart35i20b' );

% m = leaf_setproperty( m, 'allowInteraction', 1.000000 );

% m = leaf_setproperty( m, 'interactionValid', true );

% m = leaf_setproperty( m, 'gaussInfo', (unknown type ''struct'') );

% m = leaf_setproperty( m, 'stitchDFs', [] );

% m = leaf_setproperty( m, 'D', (36 values) );

% m = leaf_setproperty( m, 'C', (36 values) );

% m = leaf_setproperty( m, 'G', (6 values) );

% m = leaf_setproperty( m, 'solver', 'cgs' );

% m = leaf_setproperty( m, 'solvertolerance', 0.001000 );

% m = leaf_setproperty( m, 'diffusiontolerance', 0.000010 );

% m = leaf_setproperty( m, 'maxIters', 40.000000 );

% m = leaf_setproperty( m, 'maxsolvetime', 1000.000000 );

% m = leaf_setproperty( m, 'cgiters', 52.000000 );

% m = leaf_setproperty( m, 'simsteps', 0.000000 );

% m = leaf_setproperty( m, 'stepsperrender', 0.000000 );

% m = leaf_setproperty( m, 'growthEnabled', true );

% m = leaf_setproperty( m, 'diffusionEnabled', true );

% m = leaf_setproperty( m, 'makemovie', 0.000000 );

% m = leaf_setproperty( m, 'moviefile', '' );

% m = leaf_setproperty( m, 'codec', 'None' );

% m = leaf_setproperty( m, 'autonamemovie', true );

% m = leaf_setproperty( m, 'overwritemovie', false );

% m = leaf_setproperty( m, 'framesize', (3 values) );

% m = leaf_setproperty( m, 'mov', [] );

% m = leaf_setproperty( m, 'jiggleProportion', 1.000000 );

% m = leaf_setproperty( m, 'cvtperiter', 0.200000 );

% m = leaf_setproperty( m, 'boingNeeded', false );

% m = leaf_setproperty( m, 'initialArea', 0.940756 );

% m = leaf_setproperty( m, 'bendunitlength', 0.969926 );

% m = leaf_setproperty( m, 'targetRelArea', 1.000000 );

% m = leaf_setproperty( m, 'defaultinterp', 'min' );

% m = leaf_setproperty( m, 'readonly', false );

% m = leaf_setproperty( m, 'projectdir', 'C:\Documents and Settings\GMD\Mes documents\Le Garrec\heart morpho\Coen\GFToolbox\Heart_models' );

% m = leaf_setproperty( m, 'modelname', 'Heart35i20b' );

% m = leaf_setproperty( m, 'allowsave', 1.000000 );

% m = leaf_setproperty( m, 'addedToPath', true );

% m = leaf_setproperty( m, 'bendsplit', 0.300000 );

% m = leaf_setproperty( m, 'dorsaltop', true );

% m = leaf_setproperty( m, 'comment', '' );

% m = leaf_setproperty( m, 'bioAsplitcells', 1.000000 );

% m = leaf_setproperty( m, 'bioApullin', 0.142857 );

% m = leaf_setproperty( m, 'bioAfakepull', 0.202073 );

% m = leaf_setproperty( m, 'coderevision', 3530 );

% m = leaf_setproperty( m, 'coderevisiondate', '2011-05-10 15:51:48.530599' );

% m = leaf_setproperty( m, 'modelrevision', 0.000000 );

% m = leaf_setproperty( m, 'modelrevisiondate', '' );

% m = leaf_setproperty( m, 'vxgrad', (108 values) );

% m = leaf_setproperty( m, 'lengthscale', 1.500000 );

% m = leaf_setproperty( m, 'performinternalrotation', false );

% m = leaf_setproperty( m, 'legendTemplate', '%T: %q\n%m' );

% m = leaf_setproperty( m, 'relativepolgrad', false );

% m = leaf_setproperty( m, 'userpolarisation', false );

% m = leaf_setproperty( m, 'usepolfreezebc', false );

% m = leaf_setproperty( m, 'starttime', 0.000000 );

% m = leaf_setproperty( m, 'displayedMulti', [] );

% m = leaf_setproperty( m, 'splitmorphogen', '' );

% m = leaf_setproperty( m, 'scalebarvalue', 0.000000 );

% m = leaf_setproperty( m, 'rectifyverticals', false );

% m = leaf_setproperty( m, 'flattenforceconvex', true );

% m = leaf_setproperty( m, 'defaultazimuth', -45.000000 );

% m = leaf_setproperty( m, 'defaultelevation', 33.750000 );

% m = leaf_setproperty( m, 'defaultroll', 0.000000 );

% m = leaf_setproperty( m, 'defaultViewParams', (unknown type ''struct'') );

% m = leaf_setproperty( m, 'interactive', false );

% m = leaf_setproperty( m, 'RecordMeshes', (unknown type ''struct'') );

% m = leaf_setproperty( m, 'thicknessMode', 'scaled' );

% m = leaf_setproperty( m, 'usefrozengradient', true );

% m = leaf_setproperty( m, 'perturbInitGrowthEstimate', 0.000010 );

% m = leaf_setproperty( m, 'perturbRelGrowthEstimate', 0.010000 );

% m = leaf_setproperty( m, 'perturbDiffusionEstimate', 0.000100 );

% m = leaf_setproperty( m, 'resetRand', false );

% m = leaf_setproperty( m, 'unitbulkmodulus', true );

% m = leaf_setproperty( m, 'bioApresplitproc', '' );

% m = leaf_setproperty( m, 'bioApostsplitproc', '' );

% m = leaf_setproperty( m, 'canceldrift', false );

% m = leaf_setproperty( m, 'solverprecision', 'double' );

% m = leaf_setproperty( m, 'solvertolerancemethod', 'norm' );

% m = leaf_setproperty( m, 'allowsparse', true );

% m = leaf_setproperty( m, 'savedrunname', '' );

% m = leaf_setproperty( m, 'savedrundesc', '' );

end
