# Supplementary material for: A predictive model of asymmetric morphogenesis from 3D reconstructions of mouse heart looping dynamics
Source: eLife. 2017 Nov 28;6:e28951. doi: 10.7554/eLife.28951 (PMC5705212; doi:10.7554/eLife.28951)
Supplement: Transparent reporting form [file elife-28951-transrepform.docx]

***eLife’s* transparent reporting form Le Garrec et al., “A predictive model of asymmetric morphogenesis from 3D reconstructions of mouse heart looping dynamics”**

We encourage authors to provide detailed information *within their submission* to facilitate the interpretation and replication of experiments. If you have any questions, please contact us: [editorial@elifesciences.org](mailto:editorial@elifesciences.org).

**Sample-size estimation**

- You should state whether an appropriate sample size was computed when the study was being designed
- You should state the statistical method of sample size computation and any required assumptions
- If no explicit power analysis was used, you should describe how you decided what sample (replicate) size (number) to use

Please outline where this information can be found within the submission (e.g., page numbers or figure legends), or explain why this information doesn’t apply to your submission:

Considering a balance between experimental constraints and the chosen effect size, our experimental design provided a minimum of 2 embryos for immunofluorescence imaging after CUBIC clearing (effect size 25%, Fig. 5G), 3 embryos for HREM imaging in wild-type mouse lines (effect size 100%, Fig. 2C, 3F, 4C and 4 figure supplement 1B), 9 embryos for DiI cell labeling experiments (effect size 50%, Fig. 5E) and 5 embryos for HREM imaging in the *Shh^+/-^* mouse line (effect size 25%, Fig. 9F, G and K). The post-hoc check confirmed a minimum power of 80% for bilateral Student tests with a Type 1 error probability of 5%, using the PS software. This information is available in the Materials and Methods.

**Replicates**

- You should report how often each experiment was performed
- You should include a definition of biological versus technical replication
- The data obtained should be provided and sufficient information should be provided to indicate the number of independent biological and/or technical replicates
- If you encountered any outliers, you should describe how these were handled
- Criteria for exclusion/inclusion of data should be clearly stated
- High-throughput sequence data should be uploaded before submission, with a private link for reviewers provided (these are available from both GEO and ArrayExpress)

Please outline where this information can be found within the submission (e.g., page numbers or figure legends), or explain why this information doesn’t apply to your submission:

All sample numbers indicated in the text refer to biological replicates, i.e. different embryos. No outlier was excluded from the data analysis. For DiI cell labeling experiments, embryos that showed widespread background heart fluorescence or appeared morphologically abnormal at the end of the culture, were discarded (see Materials and Methods).

**Statistical reporting**

- Statistical analysis methods should be described and justified
- Raw data should be presented in figures whenever informative to do so (typically when N per group is less than 10)
- For each experiment, you should identify the statistical tests used, exact values of N, definitions of center, methods of multiple test correction, and dispersion and precision measures (e.g., mean, median, SD, SEM, confidence intervals; and, for the major substantive results, a measure of effect size (e.g., Pearson's r, Cohen's d)
- Report exact p-values wherever possible alongside the summary statistics and 95% confidence intervals. These should be reported for all key questions and not only when the p-value is less than 0.05.

The statistical tests used are explained in the Materials and Methods section ; the number of observations and p-value are provided in the Figure legends or in the Result section. Raw data are presented for the analysis of cell ingression at the venous pole (Figure 5E and G), the comparison between *Shh^-/-^* mutants and controls (Figures 9F, G and K, figure supplements 1 and 2 and source data 1) and for comparison between GM6001 treatment and controls (Figure 10D). When relevant, data are shown as mean and associated standard deviation, as specified in the figure legends.

Please outline where this information can be found within the submission (e.g., page numbers or figure legends), or explain why this information doesn’t apply to your submission:

(For large datasets, or papers with a very large number of statistical tests, you may upload a single table file with tests, Ns, etc., with reference to page numbers in the manuscript.)

**Additional data files (“source data”)**

- We encourage you to upload relevant additional data files, such as numerical data that are represented as a graph in a figure, or as a summary table
- Where provided, these should be in the most useful format, and they can be uploaded as “Source data” files linked to a main figure or table
- Include model definition files including the full list of parameters used
- Include code used for data analysis (e.g., R, MatLab)
- Avoid stating that data files are “available upon request”

Please indicate the figures or tables for which source data files have been provided:

For the comparison between *Shh^-/-^* mutants and controls, raw data are presented in Figures 9F, G and K, figure supplements 1 and 2 and source data 1. For the computer simulations, source codes 1 to 5 are provided in a Matlab format, including reference to the associated figures. The full list of parameters is described in the Materials and Methods section. For data analysis, tests were performed with either Excel or R statistical packages, as specified in the Materials and Methods section.
